# Oesophageal IGRT considerations for SBRT of LA-NSCLC: barium-enhanced CBCT and interfraction motion

**DOI:** 10.1186/s13014-021-01946-8

**Published:** 2021-11-14

**Authors:** Katrina Woodford, Vanessa Panettieri, Jeremy D. Ruben, Sidney Davis, Trieumy Tran Le, Stephanie Miller, Sashendra Senthi

**Affiliations:** 1 Alfred Health Radiation Oncology, The Alfred, 55 Commercial Road, Melbourne, VIC 3004 Australia; 2 Department of Surgery, Central Clinical School, Monash University, Melbourne, VIC Australia; 3 Department of Medical Imaging and Radiation Sciences, Monash University, Clayton, VIC Australia

**Keywords:** Oesophagus, Barium, Contrast, IGRT, Interfraction motion, CBCT, SBRT

## Abstract

**Background:**

To determine the optimal volume of barium for oesophageal localisation on cone-beam CT (CBCT) for locally-advanced non-small cell lung cancers (NSCLC) and quantify the interfraction oesophageal movement relative to tumour.

**Methods:**

Twenty NSCLC patients with mediastinal and/or hilar disease receiving radical radiotherapy were recruited. The first five patients received 25 ml of barium prior to their planning CT and alternate CBCTs during treatment. Subsequent five patient cohorts, received 15 ml, 10 ml and 5 ml. Six observers contoured the oesophagus on each of the 107 datasets and consensus contours were created. Overall 642 observer contours were generated and interobserver contouring reproducibility was assessed. The kappa statistic, dice coefficient and Hausdorff Distance (HD) were used to compare barium-enhanced CBCTs and non-enhanced CBCTs. Oesophageal displacement was assessed using the HD between consensus contours of barium-enhanced CBCTs and planning CTs.

**Results:**

Interobserver contouring reproducibility was significantly improved in barium-enhanced CBCTs compared to non-contrast CBCTs with minimal difference between barium dose levels. Only 10 mL produced a significantly higher kappa (0.814, *p* = 0.008) and dice (0.895, *p* = 0.001). The poorer the reproducibility without barium, the greater the improvement barium provided. The median interfraction HD between consensus contours was 4 mm, with 95% of the oesophageal displacement within 15 mm.

**Conclusions:**

10 mL of barium significantly improves oesophageal localisation on CBCT with minimal image artifact. The oesophagus moves substantially and unpredictably over a course of treatment, requiring close daily monitoring in the context of hypofractionation.

**Supplementary Information:**

The online version contains supplementary material available at 10.1186/s13014-021-01946-8.

## Background

Up to 70% of patients with potentially curable locally-advanced non-small cell lung cancer (NSCLC) do not receive radical treatment and as many as 36% receive no treatment at all [1–4]. Many factors play into why this might be the case, but concerns regarding competing comorbidities, oesophageal toxicity and the logistic requirements of attending daily treatment for 6 weeks are foremost. With increasing access to stereotactic body radiotherapy (SBRT), there is an ever-increasing proportion of early stage NSCLC patients receiving radical treatment [5–7]. SBRT may have a similar impact if applied in the locally advanced setting. The safety of a hypofractionated regime of 15 × 4 Gy-fractions has been demonstrated [8–10] and is now being compared to a conventional 30-fraction regime in a phase III trial [11]. We are currently investigating the extent to which hypofractionation can be safely achieved (ACTRN12619001186145) [12]. To accomplish this requires the most accurate image guidance possible to account for the daily position of organs-at-risk.

One of the key dose-limiting organs-at-risk when irradiating locally-advanced NSCLC is the oesophagus. Conventional fractionations with concurrent chemotherapy can result in ≥ Gr. 3 oesophagitis rates of 5–18% [13, 14], whilst hypofractionated regimes pose a greater risk of severe and even fatal reaction [10, 15]. The day-to-day position of the oesophagus can be inconsistent and is not fully accounted for during simulation and treatment planning [16–18]. Additionally, with standard cone-beam computed tomographic (CBCT) imaging, the visibility of the oesophagus is poor due its small size and similar radiographic density to the adjacent tissues. With a narrow therapeutic index, the oesophagus represents a significant obstacle in utilising SBRT for targets within the mediastinum [19, 20].

We have previously investigated the use of oral contrast with thoracic CBCT and compared 50 ml each of Gastrografin and Barium Sulfate [21]. Barium allowed better visualisation of the oesophagus on CBCT, however its density and volume led to artifacts which potentially impaired IGRT for other structures including the tumour itself. Qiu et al., using an even larger volume of barium, quantified interfractional oesophageal movement in relation to bony anatomy, and found this to be as high as 36 mm [18]. The aim of this work was to determine the optimal volume of barium required to maintain oesophageal visibility and minimise imaging artifacts. Additionally, we aimed to quantify the interfraction motion of the oesophagus relative to the tumour, an essential IGRT consideration in applying SBRT for locally-advanced NSCLC.

## Methods

### Patients and imaging

NSCLC patients were prospectively recruited into this institutional ethics approved study after providing informed written consent. Patients were included if they were being planned for a radical course of treatment for disease that extended into the mediastinum and/or hilum in which the oesophagus was a primary organ at risk.

Undiluted barium sulfate (Liquibar 62.5 %w/w, MCI Forrest) was administered just prior to acquisition of the patient’s planning 4DCT and prior to patient setup on the treatment couch for every alternate weekly CBCT. 4DCT scans were acquired in free breathing with contouring and dosimetry performed on the reconstructed average intensity projection dataset. The barium was contoured in the treatment planning system and the density was overridden and assigned a Hounsfield Unit of zero. This was to account for the institution’s treatment planning algorithm’s inability to accurately account for very high densities (Analytical Anisotropic Algorithm, Eclipse, v13.6, Varian Medical Systems, Palo Alto, CA). The variation in dose was estimated to be insignificant, due to the small size of the oesophagus compared to the treatment area and the multi-field, intensity modulated nature of the treatment plans. CBCTs were acquired in free breathing using thorax-specific protocols either in full rotation (360°, half-fan mode, 45 cm field-of-view) or half rotation “spotlight” mode (200°, full-fan mode 25 cm field-of-view) and reconstructed to 2–2.5 mm slice thickness (Varian Clinac iX; 110 kVp or Varian TrueBeam; 125 kVp). Patients were sequentially assigned to 4 barium dose levels: 25 mL, 15 mL, 10 mL and 5 mL. Barium was administered orally immediately prior to lying on the treatment couch. The time between barium administration and CBCT acquisition was recorded.

### Oesophageal visibility

**Fig. 1:**
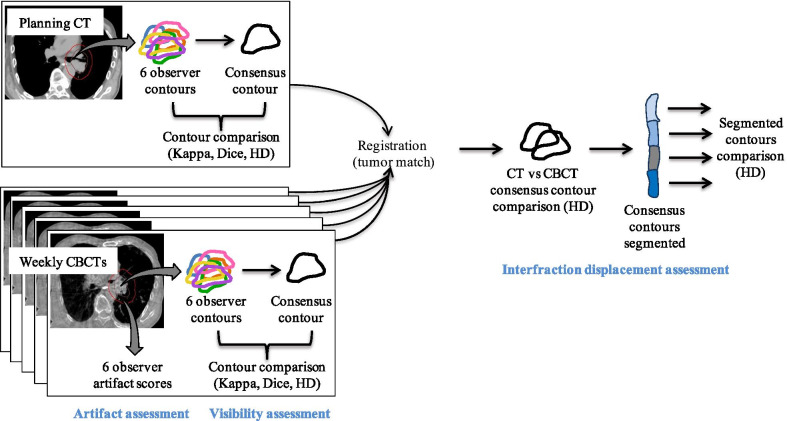
Study design flowchart

We assumed visualisation of the oesophagus during IGRT would correlate directly and objectively with manually generated contours. Hence, we utilised interobserver contouring reproducibility as a surrogate for oesophageal visualisation. The oesophagus was contoured on each planning CT and CBCT by six observers consisting of three radiation oncologists and three SBRT-trained radiation therapists. They were instructed to contour the oesophagus from 1 cm above to 1 cm below the planning target volume (PTV). Window levelling was up to the discretion of the observer as they would have during IGRT. All contouring was performed in the Aria Contouring Workspace (v13.6, Varian Medical Systems, Palo Alto, CA) with 0.1 cm resolution. The datasets were then transferred to the Computational Environment for Radiotherapy Research (CERR) software program [22] where a consensus contour was generated from the six observer contours using the simultaneous truth and performance level estimation (STAPLE) method [23]. This contour was then considered the ground truth for each dataset. Each observer contour was then compared to the consensus contour to determine the variation between them (see study flowchart in Fig. 1). How well the observers were able to visualise the oesophagus was thereby determined by how similar their contours were to the consensus contour. This was carried out separately using data from CBCTs with and without barium and the comparison was considered the improvement in visibility with barium.

### Interfraction oesophageal motion

Once a consensus contour was created for each planning CT and CBCT, the datasets were reimported to Aria. In the Image Registration Workspace each barium CBCT was rigidly registered with the planning CT based off a soft tissue tumour match and the consensus contours were then compared. We considered this difference to be due to variation in the oesophageal position over the course of treatment in relation to the tumour. Each consensus contour was then segmented into four sections to assess for trends in oesophageal motion based on anatomical levels. These sections were cervical (cricoid cartilage to sternal notch), upper (sternal notch to carina), middle (carina to midpoint between carina and gastroesophageal junction (GEJ)) and lower (midpoint between carina and GEJ to GEJ).

### Contour analysis

The contours were compared using three metrics standardly used to compare differences in contours [24]; a kappa statistic [25], a dice coefficient [26] and a Hausdorff Distance (HD)(millimetres) [27]. A summary of these metrics and their use in this study can be found in Additional file 1: Table S1. The kappa statistic was calculated in CERR, whilst the dice coefficient and HD were calculated in the SlicerRT extension of the 3D Slicer software program [28]. The interobserver contouring reproducibility assessments utilised all three metrics. The interfraction motion assessments used the HD only as the primary objective of this analysis was to understand the gross difference in the oesophagus position in the context of daily online matching and its potential dosimetric implications.

### Image artifact assessment

A qualitative assessment of contrast-induced image artifact was carried out. Each of the 6 observers scored the impact of any artifact on their ability to identify the gross tumour volume (GTV), trachea, main bronchi and central vessels on each CBCT dataset. Scores were defined as: 1 = no impact, 2 = some impact, but IGRT still achievable, 3 = Significant impact, unable to identify sufficiently for IGRT purposes. Observers were not blinded to contrast volumes, however assessments were done throughout the study period over many months making bias less likely.

### Statistical analysis

The median kappa, dice coefficient and HD were compared between CBCTs with and without contrast using a Mann–Whitney *U* test. Differences between contrast dose levels were compared using a Kruskall Wallis test with post hoc Dunn-Bonferroni pairwise comparisons. Image artifact scores were reported using descriptive statistics. Correlation between the improvement of interobserver contouring reproducibility metrics gained with barium and the non-barium metrics, contouring reproducibility and time from contrast administration, and interfraction oesophageal displacement and PTV volume or overlap volume of PTV with oesophagus were carried out using a Pearson’s correlation. Differences in interfraction oesophageal displacement between categorical variables (primary tumour laterality, thoracic nodal station involvement, involvement of mediastinal nodes, oesophagus segment) were compared using a Mann–Whitney *U* test. Statistical analysis was carried out in SPSS Statistics (IBM Corp. v26.0. Armonk, NY). Significance for all tests was deemed as *p* < 0.05.

## Results

### Patient characteristics

Of the 20 patients recruited, two patients chose to withdraw from the study—one could not tolerate barium at simulation and the other felt unwell in the days after simulation and attributed this to barium. Patient disease and treatment details are summarised in Table 1*.* Most patients had right-sided (n = 10), stage III disease (IIIA = 8, IIIB = 5, IIIC = 1) and received a median of 30 fractions (range: 15–30). Two patients had small amounts of pleural effusion, one patient had atelectasis and one patient had organising pneumonia at the start of treatment. The median PTV volume was 374.9 cc and contoured oesophagus length was 12 cm. 17 planning CTs and 90 CBCTs (44 with contrast, 46 without) were available for contouring and a total of 642 oesophagus structures were contoured by the 6 observers. One patient did not receive barium prior to their planning CT and so that dataset was not used for analysis.

**Table 1:** Patient disease and treatment details

Patient	Sex	Age	TNM	Stage	Laterality	Involved nodal stations	PTV volume (cc)	Overlap volume of PTV with oesophagus (cc)	Contoured oesophagus length (cm)	No. of fractions	No. of CBCTs with barium	No. of CBCTs without barium	Barium dose (mL)
1	F	78	T4N2M0	IIIB	L	4L, 3A	1609.9	5.96	14	30	3	3	25
2	M	68	T4N2M0	IIIB	R	2R, 4R, 7	406.4	0	12.3	30	2	2	25
3	M	84	T3N2	IIIB	R	2R, 4R, 10R	204.2	0.02	11.6	30	3	3	25
4	F	52	T2bN1M0	IIB	R	4R, 11R	337.4	0.6	8.6	30	3	3	25
5	F	54	T1N2M0	IIIA	L	4R, 4L, 5, 6, 7, 10L	343.3	5.86	10.3	30	3	3	25
6	F	67	TXN2M0	IIIA	R	7	326.3	14.2	14.8	30	3	3	15
7	F	71	T1bN2M0	IIIA	L	5, 7,10L,	231	13.27	12.8	20	2	2	15
8	M	72	T3N2M0	IIIB	L	5	513.8	8.02	11	25	2	2	15
9	M	82	T4N2M0	IIIB	R	10R	1002.9	7.83	10.8	30	3	3	15
10	M	82	T2aN2M0	IIIA	R	4R, 7, 8R, 10R, 11R	481.1	5.24	14.5	20	2	2	15
11	F	78	T3N2M1b	IVA	L	5, 10L	209	3.44	11.3	25	2	3	10
12	M	78	T4N3M1b	IVA	R	2L, 2R, 3A, 4R, 10R	548.9	4.52	15.4	15	1	2	10
13	M	85	T2bN2M0	IIIA	R	2R, 4R, 10R	485.5	1.65	13.7	30	3	2	10
14	M	54	T4N1M0	IIIA	R	10R	1353.2	17.63	17.3	20	2	2	10
15	M	76	T4N1M0	IIIA	L	10L	228.8	0.08	10.8	20	2	2	10
16	M	51	T3N3M0	IIIC	R	1L, 7	532.1	19.71	13.8	30	2	3	5
17	F	75	T2aN0M0/T1bN0M0*	IB/IA	L	–	146.4	0	9.33	30	3	3	5
18	M	55	T4N0M0	IIIA	M	–	283.42	8.42	10	30	3	3	5

^*^Two synchronous tumours, one in hilum. L = left, R = right, M = mediastinum

### Oesophageal visibility

**Fig. 2:**
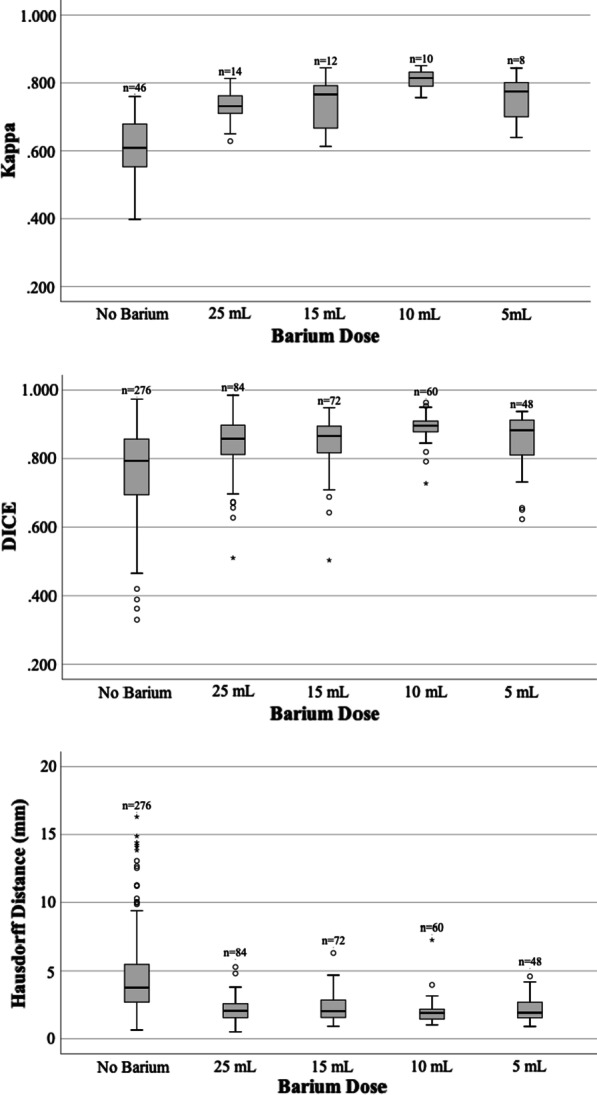
Boxplots summarising the Kappa, Dice and Hausdorff Distance metrics per barium dose level. *A higher Kappa and Dice score and a smaller Hausdorff Distance represents better reproducibility between observer contours. Outlier at HD = 29 mm in No Barium group not depicted as out of scale

Table 2 summarises the median and interquartile range of each metric for the planning CT datasets and the CBCT datasets. Barium significantly improved interobserver contouring reproducibility on CBCTs across all three metrics compared to CBCTs without barium. A significant difference between barium dose levels was found for the kappa statistic and dice coefficient, with the pairwise comparison showing a significant benefit only in the 10 mL level. There was no difference in the HD between dose levels. These results are also demonstrated in the boxplots of each metric grouped by dose level in Fig. 2.

**Table 2:** Summary of the median [IQR] Kappa, Dice and HD on the planning CTs and CBCTs

	Planning CTs (with Barium) (n = 17)	CBCTs without Barium (n = 46)	CBCTs with Barium (n = 44)	*p*-value*	CBCTs with Barium				
					25 mL (n = 14)	15 mL (n = 12)	10 mL (n = 10)	5 mL (n = 8)	*p*-value *
Kappa	0.851 [0.037]	0.607 [0.162]	0.772 [0.096]	**< 0.001**	0.732 [0.061]	0.766 [0.132]	0.814 [0.043]	0.775 [0.112]	**0.008**
Dice	0.916 [0.039]	0.791 [0.161]	0.879 [0.071]	**< 0.001**	0.859 [0.085]	0.866 [0.077]	0.895 [0.030]	0.883 [0.102]	**< 0.001**
HD (mm)	1.3 [0.5]	3.9 [2.6]	2.0 [1.0]	**< 0.001**	2.1 [1.1]	2.0 [1.3]	1.9 [0.7]	1.9 [1.2]	0.220

					*p*-values for Dunn–Bonferroni pairwise comparisons*				
					Kappa		Dice		HD
			25–15 mL		1.000		1.000		1.000
			25–10 mL		**0.006**		< **0.001**		1.000
			25–5 mL		1.000		1.000		1.000
			15–10 mL		0.051		< **0.001**		1.000
			15–5 mL		1.000		1.000		1.000
			10–5 mL		0.249		**0.036**		1.000

^*^P-values from the Mann–Whitney test (CBCTs—Barium vs. no Barium) and Kruskall–Wallis test (between Barium dose levels) with post hoc analysis

**Fig. 3:**
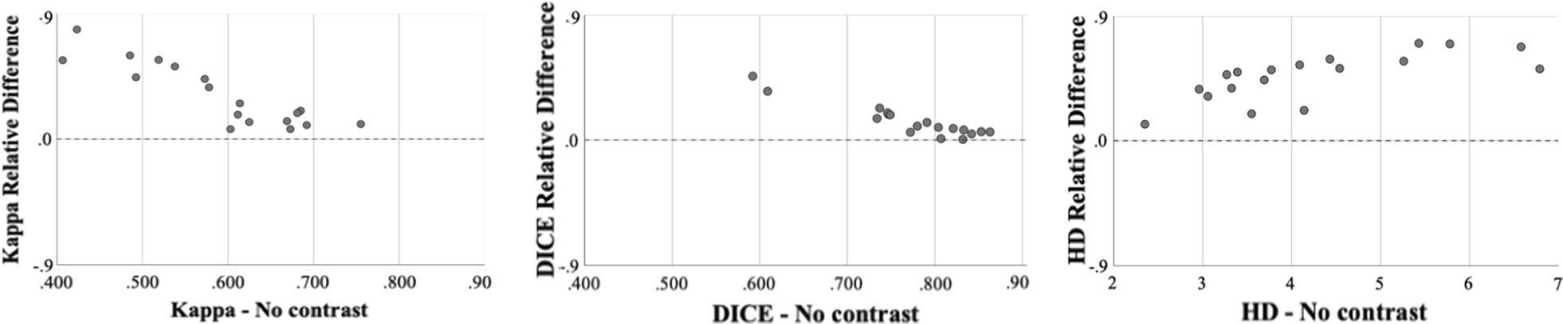
Relative difference in median Kappa, Dice and HD on barium CBCTs compared to non-contrast CBCTs. A positive difference refers to an improvement in the metric gained by barium use. Each dot represents a patient

The amount of improvement in interobserver contouring reproducibility gained with barium per patient correlated with the non-contrast CBCT metrics. In summary, the poorer the contouring reproducibility on the non-contrast CBCT the greater the improvement the barium provided. The metric improvements gained by each patient are displayed in Fig. 3. For the Dice coefficient and Kappa statistic there was a very strong (r =  − 0.909, *p* < 0.001) and strong (r =  − 0.807, *p* < 0.001) negative correlation respectively between the improvement gained and the baseline reading for either metric, whilst the HD demonstrated a very strong positive correlation (r = 0.921, *p* < 0.001).

Two patients did not have time measurements for analysis. Of those that did the median time between contrast administration and CBCT acquisition was 6 min (2–16 min). All CBCTs except two were acquired within 10 min of contrast consumption, with the largest time of 16 min occurring due to a delay caused by equipment breakdown. No correlation between the contour comparison metrics and time was found (Kappa r = 0.168, *p* = 0.306, Dice r = 0.268, *p* = 0.099, HD r =  − 0.024, *p* = 0.883). The median kappa statistic and HD were significantly higher in datasets acquired using the half rotation “spotlight” protocol compared to the full rotation protocol (Kappa 0.790 vs. 0.750, *p* = 0.018, Dice 0.884 vs. 0.860, *p* = 0.072 and HD 1.8 vs. 2.2 mm, *p* = 0.009).

### Contrast-induced image artifact

The scoring of detrimental artifact was variable across the dose levels. Of the 1,056 total scores recorded 4%, 10%, 6% and 8% were scores of 3 in the 25 mL, 15 mL, 10 mL and 5 mL dose levels respectively. This compares to 12% found in our previous study using 50 mL of barium [21]. Of the 71 scores of 3 recorded, the majority were for central vessels (39%), followed by GTV (30%), Bronchi (27%) and Trachea (4%). A score of 3 was more common in some patients than in others, with 39% having no scores of 3 recorded and 61% having no scores of 3 for the GTV. A moderate positive correlation was found between the average GTV artifact score and the percentage of the PTV overlapping with the oesophagus (r = 0.527, *p* = 0.025). Of the 7 patients with a score of 3 for the GTV, 6 of these showed marked improvements in each of their 3 contouring reproducibility metrics. The perception of the artifact also appeared to be observer dependent. 100%, 46%, 64% and 63% of scores of 3 came from the same observer in the 25 mL, 15 mL, 10 mL and 5 mL dose levels, while another observer did not score any region of interest on any dataset with a 3.

### Interfraction oesophageal displacement

Comparing the consensus contours between the barium CBCTs and the planning CTs, 69% of patients had a reduction in oesophagus volume by an average of 4.2 cc in the first 2–3 weeks of treatment and half of those saw a further reduction in week 4. Of those patients who had a barium CBCT in weeks 5 or 6, 63% had a reduced volume compared to the planning CT.

**Fig. 4:**
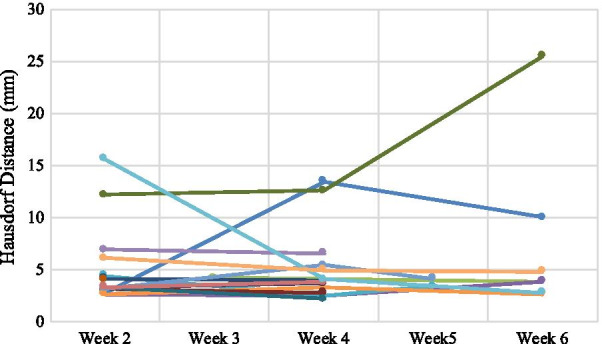
Gross oesophageal displacement between planning CT and barium CBCT over the treatment course. Each line/colour represents a patient

After registering each barium CBCT with the planning CT, the median HD between the consensus contours was 3.9 mm (range 2.2–25.5 mm). Figure 4 shows the variation of these HD measurements (planning CT vs. CBCTs) for each patient over the course of treatment, noting the HD provides magnitude and not direction of displacement. 76% of the variation was within 5 mm suggesting a systematic difference, potentially due to the use of CBCT as opposed to the planning CT. Despite this trend larger, more random, deviations were observed for some patients and fractions. 14% and 5% of the measurements were over 10 mm and 15 mm respectively. There was no significant difference in median HD between the four anatomical segments of the oesophagus (cervical 3.0 mm, upper 3.7 mm, middle 3.2 mm, lower 3.6 mm, *p* = 0.135). No correlation was found between the HD and primary tumour laterality (right 3.9 mm, left 3.4 mm, *p* = 0.862) or the volume of PTV overlapping the oesophagus (r = 0.050, *p* = 0.751). A weak correlation was seen between oesophagus displacement and PTV volume (r = 0.392, *p* = 0.010). There was no significant difference between displacement and any of the involved individual lymph node stations. A significantly smaller HD was recorded in patients with mediastinal lymph node involvement than in those without (3.4 mm vs. 5 mm, *p* = 0.008).

## Discussion

We have demonstrated that a small amount of barium is sufficient in improving the interobserver contouring reproducibility of the oesophagus on CBCT imaging with no improvement with increasing dose. Barium improved the contouring reproducibility metrics from baseline for each patient. The amount of improvement the contrast provided varied between patients and was less dose dependent than it was patient dependent. Improvement correlated with the baseline measurement, indicating the poorer the interobserver contouring reproducibility without contrast, the greater the improvement the contrast provided. From these results we infer that the improved precision in observer contouring corresponds to improved oesophageal visibility due to barium administration. The only dose level that showed a significant improvement over the others was 10 mL, although this was found in the Kappa and Dice metrics and not the HD. Previous reports have shown that the Dice and HD do not correlate and instead complement one another [29], highlighting the importance of using a combination of metrics in studies like this [24].

**Fig. 5:**
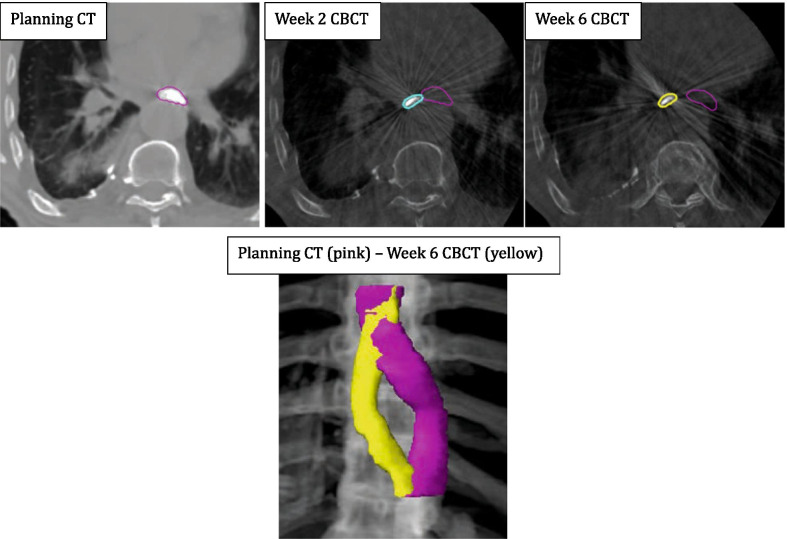
Variation in oesophageal position from planning CT to week 2 CBCT and week 6 CBCT of one patient

There is growing evidence to show that hypofractionated, SBRT-like treatments may play a significant role in patients with locally-advanced NSCLC who are ineligible for concurrent chemoradiotherapy [8–11, 30]. These regimes can cause severe toxicity to central organs like the oesophagus. The oesophagus is mobile and its position is influenced by both normal physiological functions (peristalsis, swallowing, respiration, cardiac rhythm) and tumour-related changes (tumour regression/progression, atelectasis, plural effusion) [16, 31]. Its position cannot be inferred by bony landmarks, nor assumed to be consistent over a course of radiotherapy spanning weeks, hence monitoring its daily position is paramount. We found the median interfraction oesophagus HD was 4 mm compared to the planning CT with 14% measuring over 10 mm. The magnitude of displacement could not be predicted by anatomical level, primary tumour laterality, overlap of the PTV with the oesophagus, nor by involvement of specific nodal stations. No patients in this study had significant tumour changes that required re-simulation, however reports have shown this can occur in 20% of locally-advanced lung cancer patients with significant risk to mediastinal structures if not actioned upon [32]. Figure 5 demonstrates a patient from this study with extreme variation in oesophageal position from as early as week 2 through to week 6. It could be argued that the introduction of an oral contrast agent to the oesophageal lumen may induce oesophageal motion through peristalsis. Given the small volume of barium used, the rapid nature of oesophageal transit times (seconds) [33] and the timeframe from administration to CBCT (minutes) this is unlikely to be a significant consideration. We also found the oesophageal volume decreased over the treatment course in most patients. We speculate that this is due to treatment related oesophagitis, causing constriction and luminal narrowing [34]. This then results in less barium accumulating in the lumen and as the external boundary of the lumen can be difficult to see on CBCT, the contoured volumes have shrunk.

Qiu et al. [18] have also recently reported on the use of barium with pre-treatment CBCTs for this purpose. They utilised 30 mL of barium before and a further 30 mL during CBCT acquisition and found oesophageal motion was greater in the left–right direction than the anterior–posterior direction, more prominent in the middle oesophagus and in patients with right sided disease or with bulky mediastinal lymph nodes. However, these positional variations were reported with reference to the bony landmarks. It is well understood that thoracic tumours move independent of bones [35, 36] and so in the context of hypofractionated SBRT treatments online soft tissue match to tumour is preferred [37]. We thus sought in this study to understand the relationship of the oesophageal position in relation to the tumour position over the course of treatment to help guide dosimetric decisions during treatment planning. The addition of barium to the planning and treatment of these patients can also help guide and verify the position of high dose gradients, for example when utilising a contralateral oesophageal-sparing technique [38, 39].

The consequence of barium-induced image artifact on CBCT has not been investigated until now. Artifact perception was not dose dependent as we had hypothesized, but instead influenced by individual patient and tumour anatomy. Proximity of organs/targets to the oesophagus had a greater impact than barium dose. Artifact influenced visibility of the central vessels more than other organs, which is acceptable given they are not as dose-restricting and their position is consistent. It remains of concern that some observers perceived the artifact to impede their ability to visualise the GTV. The more the PTV overlapped with the oesophagus, the greater the chance the barium influenced one's ability to clearly see the GTV on CBCT. However, in nearly all of these patients the oesophageal visibility was greatly improved by the barium, so a cost–benefit question arises in some patients between oesophageal and GTV visibility. Furthermore, the perception of image artifact is subjective and varies significantly between observers. In practice this subjectivity may be reduced because multiple staff are generally involved with online pre-treatment CBCT evaluation.

We found that in order to capture 95% of the oesophageal positional variations during treatment planning a planning risk volume (PRV) of 15 mm would be required. Qiu et al. [18] reported smaller 95% margins between 2.8 and 10.3 mm depending on location and direction, whilst Cohen et al. reported 8–12 mm although theirs reported from CBCTs of oesophageal cancers [40]. The limits of SBRT for locally-advanced NSCLC are not yet known and we are investigating this in a phase 1 dose escalation trial [12]. To incorporate a 15 mm PRV around the oesophagus will be impractical for most node positive NSCLC undergoing SBRT and a large PRV may jeopardise tumour control. Given a proportion of the shifts appeared random in nature (Fig. 4) the best solution may be online adaptive treatment strategies where the precise oesophageal position of the day is determined and the patient simply not treated if there is a significant shift into the high dose region. Additionally, with accurate visualisation and determination that a shift is more systematic, replanning or adaptive changes can be initiated.

This study has a number of limitations. The patient numbers are small and the results highlight the great inhomogeneity that this disease cohort presents. As the sample size per barium dose group was small, eliminating the influence of patient or disease variables on the results per dose group was impractical. Similarly, the number of treatment fractions, weekly CBCTs and patient numbers differed between groups, which may have influenced the results. Our finding of improved interobserver contouring reproducibility with barium demonstrates improved precision of oesophageal localisation. It does not necessarily, however demonstrate improved accuracy of oesophageal position. Considering the image quality of a linac CBCT (and not an MRI-linac for example) the true position of the oesophagus cannot be accurately determined. As with all radiotherapy studies drawing conclusion from human-generated contours, the resultant ground truth is dependent on the number and expertise of the observers. We intentionally chose both radiation oncologists and SBRT-trained therapists to participate as this reflects the true allocation of responsibility in the clinic. The study is further strengthened by the use of 6 observers to contour and assess image-artifact on each dataset, reducing the influence of investigator bias. Finally, in this study CBCTs were acquired weekly as per department protocol for conventionally fractionated radical treatment. Our results could have benefited had daily CBCTs been used. This study could be repeated in the future in patients receiving a hypofractionated course when daily CBCTs would be standard.

## Conclusions

Small doses of barium delivered conveniently prior to patient setup greatly improve the interobserver contouring reproducibility of the oesophagus on CBCT and do not significantly degrade the image quality. Barium has helped show that the position of the oesophagus in relation to the tumour is variable and unpredictable over a course of treatment, requiring careful dosimetric consideration, close monitoring and ideally adaptive treatment strategies.

## Supplementary Information

**Additional file 1.** Contour analysis metrics and their use in this study.

## Data Availability

The datasets used and/or analysed during the current study are available from the corresponding author on reasonable request.

## Abbreviations

4DCT: 4-Dimensional computed tomography
CBCT: Cone beam computed tomography
CERR: Computational environment for radiotherapy research
CT: Computed tomography
GEJ: Gastroesophageal junction
GTV: Gross tumour volume
HD: Hausdorff Distance
IGRT: Image-guided radiotherapy
IQR: Interquartile range
LA: Locally-advanced
NSCLC: Non-small cell lung cancer
PRV: Planning risk volume
PTV: Planning target volume
SBRT: Stereotactic body radiotherapy
STAPLE: Simultaneous truth and performance level estimation
TNM: Tumour node metastasis
