# Supplementary material for: Oesophageal IGRT considerations for SBRT of LA-NSCLC: barium-enhanced CBCT and interfraction motion
Source: Radiat Oncol. 2021 Nov 14;16:218. doi: 10.1186/s13014-021-01946-8 (PMC8591953; doi:10.1186/s13014-021-01946-8)
Supplement: Supplementary file 1 — Additional file 1. Contour analysis metrics and their use in this study. [file 13014_2021_1946_MOESM1_ESM.docx]

**Supplementary Table 1** – Contour analysis metrics

|  | Description | Output | Interpretation | Study aim |
| --- | --- | --- | --- | --- |
| Kappa Statistic (25) | A measure of agreement beyond chance in a group of contours. | -1 to +1 | ≤ 0 = no agreement  0.01-0.20 = none to slight  0.21-0.40 = fair  0.41-0.60 = moderate  0.61-0.80 = substantial  0.81-1.00 = almost perfect agreement | Inter-observer contouring reproducibility (i.e. oesophageal visibility) |
| Dice Coefficient (26) | A measure of similarity based off the overlap regions of two contours. | 0 to 1 | 0 = No overlap  1 = Complete overlap | Inter-observer contouring reproducibility (i.e. oesophageal visibility) |
| Hausdorff Distance (HD) (27) | Magnitude of gross deviation between two contours. To minimize the effect of outliers the largest distance that fell within the 95% confidence interval was used. | Distance in mm | The larger the distance the greater the variation between the contours. | Inter-observer contouring reproducibility (i.e. oesophageal visibility)  Interfraction oesophageal displacement |
